# Living donor liver transplantation can address disparities in transplant access for patients with primary sclerosing cholangitis

**DOI:** 10.1097/HC9.0000000000000219

**Published:** 2023-08-03

**Authors:** Fernanda Onofrio, Katina Zheng, Cherry Xu, Shiyi Chen, Wei Xu, Mary Vyas, Katie Bingham, Keyur Patel, Leslie Lilly, Mark Cattral, Nazia Selzner, Elmar Jaeckel, Cynthia Tsien, Aliya Gulamhusein, Gideon M. Hirschfield, Mamatha Bhat

**Affiliations:** 1 Ajmera Transplant Program, University Health Network, Toronto, Ontario, Canada; 2 Department of Medicine, University of Toronto, Toronto, Ontario, Canada; 3 Biostatistics Department, Princess Margaret Cancer Center, University Health Network, Toronto, Ontario, Canada; 4 PSC Partners Canada; 5 Division of Gastroenterology and Hepatology, Department of Medicine, University of Toronto, University Health Network, Toronto, Ontario, Canada

## Abstract

**Background:**

Liver transplantation (LT) is frequently lifesaving for people living with primary sclerosing cholangitis (PSC). However, patients are waitlisted for LT according to the model for end-stage liver disease-sodium (MELD-Na) score, which may not accurately reflect the burden of living with PSC. We sought to describe and analyze the clinical trajectory for patients with PSC referred for LT, in a mixed deceased donor/living donor transplant program.

**Methods:**

This was a retrospective cohort study from November 2012 to December 2019, including all patients with PSC referred for assessment at the University Health Network Liver Transplant Clinic. Patients who required multiorgan transplant or retransplantation were excluded. Liver symptoms, hepatobiliary malignancy, MELD-Na progression, and death were abstracted from chart review. Competing risk analysis was used for timing of LT, transplant type, and death.

**Results:**

Of 172 PSC patients assessed, 84% (n = 144) were listed of whom 74% were transplanted. Mean age was 47.6 years, and 66% were male. Overall mortality was 18.2% at 2 years. During the follow-up, 16% (n = 23) were removed from the waitlist for infection, clinical deterioration, liver-related mortality or new cancer; 3 had clinical improvement. At listing, 82% (n = 118) had a potential living donor (pLD). Patients with pLD had significantly lower waitlist and liver-related waitlist mortality (HR 0.20, *p*<0.001 and HR 0.17, *p*<0.001, respectively), and higher rates of transplantation (HR 1.83, *p* = 0.05). Exception points were granted to 13/172 (7.5%) patients.

**Conclusions:**

In a high-volume North American LT center, most patients with PSC assessed for transplant were listed and subsequently transplanted. However, this was a consequence of patients engaging in living donor transplantation. Our findings support the concern from patients with PSC that MELD-Na allocation does not adequately address their needs.

## INTRODUCTION

Primary sclerosing cholangitis (PSC) is a rare chronic cholestatic liver disease, characterized by progressive inflammatory destruction of the intrahepatic and/or extrahepatic bile ducts, leading to bile stasis, fibrosis, and ultimately secondary biliary cirrhosis and end-stage liver disease.^1–3^ The clinical presentation of PSC is often in association with inflammatory bowel disease (IBD).^4^ In adult liver transplantation (LT) practice, patients with PSC represent the youngest patient population served on average, and one of the few liver diseases remaining without any approved medical interventions. Although a heterogeneous disease in regard to clinical course, for patients referred to tertiary care centers, the average time from diagnosis to death or LT remains about 10–22 years.^5–7^ LT therefore remains essential in PSC care, a reality patients are acutely aware of from the time of diagnosis.^8^ Indications for LT in PSC include recurrent cholangitis, decompensated cirrhosis, and intractable pruritus.^9,10^ PSC patients are waitlisted for LT according to the model for end-stage liver disease-sodium (MELD-Na) score, which may not, however, adequately capture patient needs, in part because of underweighting of the bilirubin.^11^ As a consequence, many PSC patients elect to pursue living donor liver transplant (LDLT) in centers with this option available.

Exception points can be applied to a patient with PSC, who receives a baseline of 22 and a 3-point increase every 90 days up to 40 points. The criteria include 2 episodes of culture-proven bacteremia or septic complications of bacterial cholangitis or refractory pruritus. Refractory pruritus is defined as intractable pruritus secondary to underlying cholestatic liver disease and refractory, that is both intolerant to all standard medical therapies and severely impacting/impairing quality of life.^12^

LDLT is an effective, but resource-intensive alternative to deceased donor LT (DDLT) and aims to improve LT access and patient outcomes.^13^ While LDLT is a common practice in some countries such as South Korea, where it comprised over 75% of liver transplants in 2019.^14^ LDLT is much less common in countries where there is greater access to DDLT. In 2018, LDLT comprised 0.5% of transplants in the United Kingdom, 5% in the United States, and 13.6% in Canada.^15^ In a recent study at our center, transplants with LDLT and DDLT were 24% and 76%, respectively.^16^ LDLT shortens the median time to transplant and reduces the risk of dropping off the waiting list due to disease progression and death.^17^ LDLT has been demonstrated to have similar excellent outcomes compared to DDLT,^13^ despite a higher rate of surgical complications.^18^ Our study sought to analyze the clinical trajectory over time for patients with PSC once referred for LT, in a mixed deceased donor/living donor transplant program. We document real-world evidence for the high use of LDLT in our PSC population, as an inference for inadequacies of a MELD-Na allocation system.

## METHODS

### Study design

A single-center, retrospective cohort study was performed to investigate the clinical course of PSC patients referred for LT. Patients who required multiorgan transplant or retransplantation were excluded. This study was approved by the University Health Network Research Ethics Board (ID 21-5783) with a waiver of informed consent.

### Study population

All PSC patients referred for assessment at the University Health Network Liver Transplant Clinic in Toronto, Canada from November 2012 to December 2019 were included. Follow-up was until March 2021. For patients who were transplanted, the clinical diagnosis of PSC was consistent with the explant pathology.

### Variables analyzed

Relevant demographics, clinical, and biochemical data were retrieved from electronic patient records for each participant. Clinical variables included associated autoimmune diseases, hepatobiliary malignancies, pruritus, ascites, cholangitis episodes, encephalopathy, cirrhosis, and variceal bleeding. Information regarding exception points, availability of a potential living donor (pLD), type of transplant (living or deceased donor), comorbidities, and clinical outcomes were also collected. Longitudinal blood test results were retrieved from the time of referral up to date of transplant, or to date last seen if not transplanted. Pathology results from explants were also collected.

A pLD was defined as an individual who had submitted a medical history form and was being evaluated as a living donor, who was found suitable following initial screening and imaging assessments.^19^ However, they may not ultimately be deemed acceptable for donation, and only about one-third eventually proceed to surgery.^19^ A patient with a pLD may receive an LDLT, DDLT, or no transplant.

### Statistical analysis

Descriptive statistics were stratified by whether patients were listed for transplantation and whether they received exception points. Counts (proportions) were calculated for categorical variables, whereas mean (SD) and median (range) were provided for continuous variables. Group differences were compared using 2 sample *t* tests or Wilcoxon tests for continuous variables, chi-squared tests, or Fisher exact tests for categorical variables. Cumulative incidence plots of overall mortality were generated, treating transplant as a competing risk. Overall mortality was stratified by the availability of a pLD at listing, and the difference between the 2 groups was assessed using the log-rank test. In addition, cumulative incidence curve of liver-related mortality and cumulative incidence curve of transplant were plotted and stratified by pLD. The differences between patients with and without pLD were analyzed using Gray tests. Analysis was truncated once <10% of patients remained in the cohort at 2 years. For liver-related mortality, both transplant and non–liver-related mortality were treated as competing risks. For cumulative incidence of transplant, death was treated as a competing risk. Fine-Gray competing risk regression models were used to examine the associations between risk factors and outcomes, including mortality, transplant, and treatment-related mortality. Sex (male or female), age less than 60, and body mass index <25 were a priori variables used in the analysis. All statistical analyses were carried out using SAS 9.4 software (Cary, NC).

## RESULTS

### Demographics

A total of 172 patients were referred for LT assessment during the study period. Median age (at the time of listing or if not listed, age when first seen) was 47.6 years, and 65.7% were male (Table 1). In historical notes, 21/165 (12.7%) were described as having an overlap with autoimmune hepatitis (AIH), while113/165 (68.5%) had concurrent IBD. In total, 12.67% of patients (n = 19) received a colectomy with 12 (66.7%) being for colitis and 2 for cancer (11.1%).

**TABLE 1:** Characteristics of 172 PSC patients evaluated for liver transplant

	Listed (n-144)					
Clinical features	No pLD (n = 26), n (%)	pLD (n = 118), n (%)	*p*	Total listed (n = 144), n (%)	Not listed (n = 28), n (%)	Total (n = 172), n (%)
Age (median, IQR)	54.0 (41.0–61.0)	46.0 (36.0–57.0)	0.13	47.0 (37.0–58.0)	51.5 (39.5–62.0)	48.5 (37.0–58.0)
Sex (% male)	18 (61.54)	77 (65.25)	0.72	93 (64.58)	20 (71.43)	113 (65.70)
BMI (median, IQR)	26.1 (22.6–28.5)	24.3 (22.1–27.3)	0.13	24.4 (22.3–27.3)	25.6 (20.9–30.2)	24.4 (22.1–27.7)
MELD-Na at listing (median, IQR)	21.0 (15.0–28.0)	18.0 (14.0–22.0)	0.027^a^	18.0 (14.0–23.0)	NA	18.0 (14.0–23.0)
Blood type	—	—	0.40	—	—	—
A	8 (30.77)	45 (38.14)	—	53 (36.81)	6 (26.09)	59 (35.33)
B	1 (3.85)	15 (12.71)	—	16 (11.11)	1 (4.35)	17 (10.18)
AB	2 (7.69)	6 (5.08)	—	8 (5.56)	0	8 (4.79)
O	15 (57.69)	52 (44.07)	—	67 (46.53)	16 (69.57)	83 (49.70)
Associated diseases						
IBD	—	—	0.13	—	—	0.87
Crohn	2 (8.70)	19 (17.12)	—	21 (15.67)	3 (15.79)	24 (15.69)
Ulcerative colitis	9 (39.13)	61 (54.95)	—	70 (52.24)	12 (63.16)	82 (53.59)
Indeterminate	1 (4.35)	04 (3.60)	—	5 (3.73)	0 (0)	5 (3.27)
Colectomy	2 (10.53)	13 (11.61)	0.99	15 (11.45)	4 (21.05)	19 (12.67)
PSC-AIH	5 (19.23)	16 (14.15)	0.55	21 (15.11)	1 (3.85)	22 (13.33)
Clinical manifestations						
Pruritus	13 (65.00)	65 (57.02)	0.50	78 (58.21)	10 (55.56)	88 (57.89)
Cholangitis	—	—	0.24	—	—	—
0	11 (55.00)	43 (39.09)	—	54 (41.54)	4 (23.53)	58 (39.46)
1	1 (5.00)	20 (18.18)	—	21 (16.15)	2 (11.76)	23 (15.65)
>1	8 (40.00)	47 (42.73)	—	55 (42.31)	11 (64.71)	66 (44.90)
Complications						
Cirrhosis	21 (91.30)	100 (86.96)	0.74	121 (87.68)	17 (73.91)	138 (85.71)
Ascites	14 (63.64)	66 (57.89)	0.62	80 (58.82)	12 (57.14)	92 (58.60)
Encephalopathy	10 (45.45)	35 (31.25)	0.20	45 (33.58)	10 (50.00)	55 (35.71)
Esophageal varices	17 (80.95)	71 (61.21)	0.08	88 (64.23)	9 (42.86)	97 (61.39)
Variceal bleeding	5 (25.00)	21 (19.27)	0.55	26 (20.16)	3 (16.67)	29 (19.73)
Cholecystectomy	4 (21.05)	29 (26.36)	0.78	33 (25.58)	6 (37.50)	39 (26.90)
ERCP stricture dilatation	3 (21.43)	28 (35.00)	0.37	31 (32.98)	5 (45.45)	36 (34.29)
PTC	1 (4.35)	10 (8.70)	0.69	11 (8)	0 (0)	11 (7)
Cancer	—	—	0.23	—	—	—
HCC	3 (15.79)	4 (3.45)	—	7 (5.19)	0	7 (4.67)
CCA	6 (5.17)	0	—	6 (4.44)	1 (6.67)	7 (4.67)
Other	0 (0)	4 (3.45)	—	4 (2.76)	1 (6.67)	5 (3.34)
Exception points	—	—	0.99	—	—	—
Cholangitis	2 (100)	7 (63.64)	—	9 (69.23)	—	—
Pruritus	0	2 (18.18)	—	2 (15.38)	—	—
HCC	0	1 (9.09)	—	1 (7.69)	—	—
Other	0	1 (9.09)	—	1 (7.69)	—	—

*Note:* Data are presented as no. (%) unless otherwise stated.

a MELD-Na at time first seen.

Abbreviations: AIH, autoimmune hepatitis; CCA, cholangiocarcinoma; ERCP, endoscopic retrograde cholangiopancreatography; IBD, inflammatory bowel disease; MELD-Na, model for end-stage liver disease-sodium; pLD, potential living donor; PSC, primary sclerosing cholangitis; PTC, percutaneous transhepatic catheter.

In total, 93 patients had a diagnosis of cholangitis (61.5%). Overall, 44.9% had 2 or more episodes of cholangitis, 15.6% had 1 episode, and 39% did not have any reported episodes. However, only 9 patients received exception points for cholangitis. Thirty-nine patients (26.9%) had a cholecystectomy, 11 patients (7%) had a percutaneous transhepatic catheter (PTC) drain inserted, and 36 (34%) needed ERCP for stricture dilatation.

Of the total number of patients, 61.3% (n = 92) developed ascites, 35.7% (n = 55) HE, and 57.9% (n = 88) had pruritus documented in clinical notes. Only 2 patients received exception points for pruritus.

Median MELD-Na at the time of listing was 18 (range: 6–40), and 21 (range: 8–40) at the time of transplant. Out of 106 explants, 6 had HCC, and 3 had incidental cholangiocarcinoma (CCA).

### Liver transplant

**FIGURE 1:**
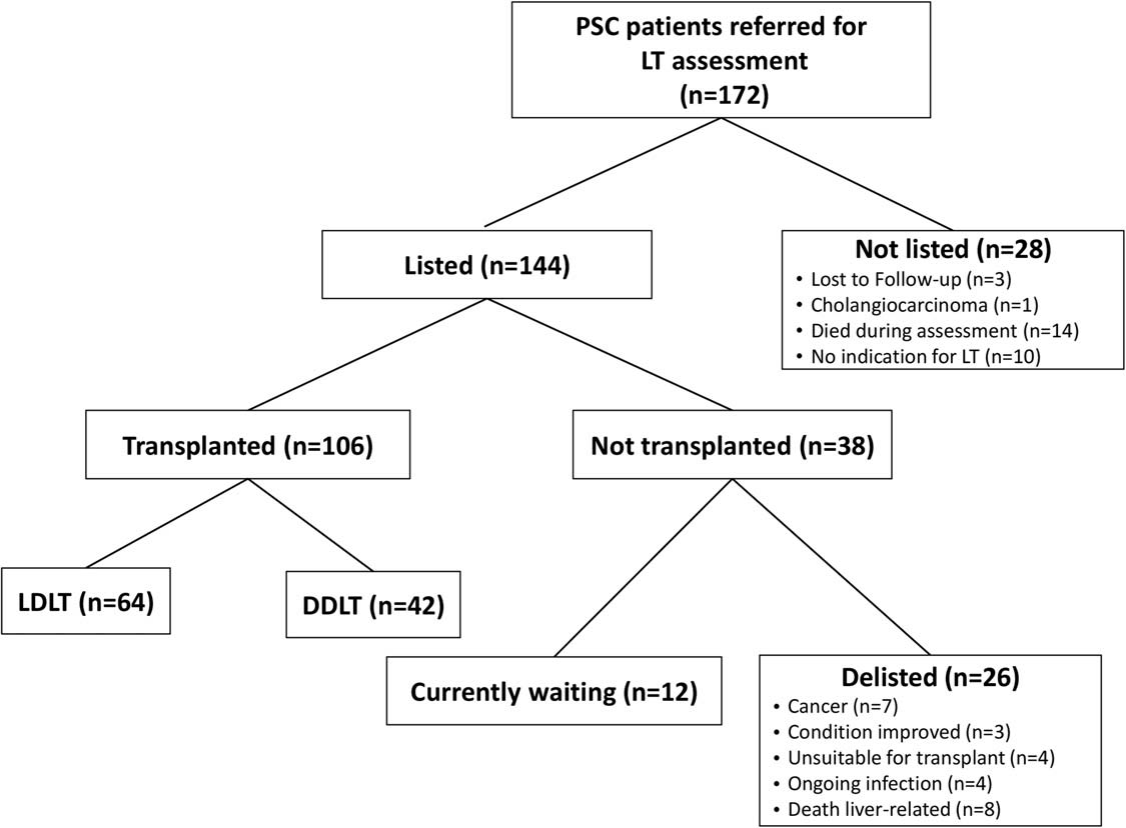
Flow chart of patients with PSC referred for LT (n = 172). Abbreviations: DDLT, deceased donor liver transplant; LDLT, living donor liver transplant; LT, liver transplantation; PSC, primary sclerosing cholangitis.

**FIGURE 2:**
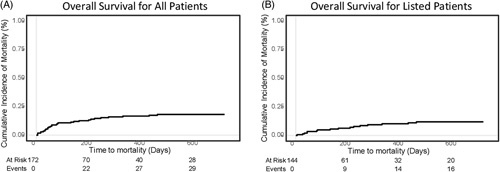
(A) Kaplan-Meier curves. (B) Overall survival (n = 172) of all patients and listed patients (n = 144), from time to listing (or date first seen if not listed) to date of death on the waitlist, date of last seen on the waitlist, or date of transplant. Transplant was treated as a competing risk. Plot is truncated at 2 years ensuring at least 10% of patients are still in the cohort.

Most patients, 144/172 (83.7%) who were assessed were listed; 106/144 (73.6%) were transplanted, and 12/144 (8.3%) were waiting for transplant at the time of our analysis (Figure. 1). Twenty-eight patients were not listed for the following reasons: lost to follow-up (n = 3), no current indication for LT (n = 10), new diagnosis of advanced CCA (n = 1), and death during LT assessment (n = 14). During follow-up (average of 2.68 y), 26/144 (18.1%) were removed from the waitlist due to ongoing infection (n = 4), clinical deterioration (n = 4), liver-related mortality (n = 8), new diagnosis of cancer (n = 7) (including 3 patients with CCA), or clinical improvement (n = 3). Overall, the cumulative incidence of mortality was 16.7% (11.8%–23.7%) at 1 year, and 18.2% (13.1%–25.4%) at 2 years (Figure 2). For patients without a pLD, the cumulative incidence of mortality was 24.9% (12.1%–51.2%) at 1 year, and 37.8% (20.6%–69.4%) at 2 years. For patients with a pLD, the cumulative incidence of mortality was 7.1% (3.6%–13.9%) at both 1 and 2 years.

### Comparisons among patients with pLD and those without pLD

Overall, 64% of females and 58% of males received LDLT. There was no significant difference between males and females (*p* = 0.55). IBD was present significantly more frequently (*p* = 0.02) in patients with pLD (85/118, 72%) than in patients without pLD (12/25, 48%).

**FIGURE 3:**
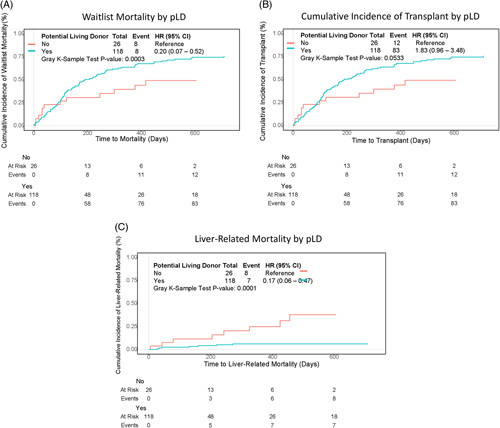
Outcomes by availability of a pLD from time to listing to date of death on the waitlist, date of last seen on the waitlist, or date of transplant. Waitlist mortality with transplant as a competing risk (*p*<0.001) (A), cumulative incidence of transplant with death as a competing risk (*p* = 0.05) (B), and liver-related mortality with transplant and non–liver-related death competing risks (*p*<0.001) (C). Plots truncated at 2 years ensuring at least 10% of patients are still in the cohort. Abbreviation: pLD, potential living donor.

Of all listed patients, 118/144 (81.9%) had a pLD at listing, of whom 94 were transplanted; 64 received LDLT (25 female and 39 male) and 30 received DDLT (14 female and 28 male) Table 2. Patients with a pLD had a significantly lower MELD-Na at listing compared with those without pLD (median 18 vs. 21, *p* = 0.03). They also had significantly lower waitlist mortality (HR 0.20, 95% CI, 0.07–0.52, *p* = 0.0003) and liver-related waitlist mortality (HR 0.17 95% CI, 0.06–0.47, *p* = 0.0001) at 2 years from listing. In addition, they were transplanted more often than those without pLD (HR 1.83, 95% CI, 0.96–3.48, *p* = 0.053) (Figure 3A–C). At the end of follow-up, fewer patients with pLD remained on the waitlist (6% vs. 19%).

**TABLE 2:** Main outcomes for listed patients by exception points and pLD status

	Exception point status		pLD status		
Outcome	Without exception points (n = 131), n (%)	With exception points (n = 13), n (%)	Without pLD (n = 26), n (%)	With pLD (n = 118), n (%)	Total listed patients (n = 144), n (%)
Transplanted	96 (73.28)	10 (76.92)	12 (46.15)	94 (79.66)	106 (73.61)
LDLT	58 (60.42)	6 (60)	0	64 (68.09)	64 (60.38)
DDLT	38 (39.58)	4 (40)	12 (100)	30 (31.91)	42 (39.62)
Time to transplant (d) (median, IQR)	122.0 (56.5–282.5)	235.5 (167.0–377.0)	68.5 (24.5–273.0)	143.0 (77.0–314.0	133.0 (63.0–300.0)
Still on waitlist	11 (8.40)	1 (7.69)	5 (19.23)	7 (5.93)	12 (8.33)
Dropped off waitlist					
Cancer	5 (3.82)	0	0	5 (4.24)	5 (3.47)
Lost to follow-up	0	0	0	0	0
Lost to follow-up and cancer	1 (0.69)	0	0	1 (0.85)	1 (0.69)
Other	3 (2.29)	0	1 (3.85)	2 (1.69)	3 (2.08)
Died on waitlist	15 (11.45)	2 (15.38)	8 (30.77)	9 (7.63)	17 (11.81)
Time to death (d) (median, IQR)	130.0 (43.0–272.0)	699.0 (219.0–1179.0)	224.0 (61.0–375.0)	130.0 (43.0–219.0)	161.0 (43.0–272.0)

*Note:* Data are presented as no. (%) unless specified.

Abbreviations: DDLT, deceased donor liver transplant; LDLT, living donor liver transplant; pLD, potential living donor.

Both males and females benefited from having a pLD, which resulted in significantly decreased waitlist and liver-related waitlist mortality at 2 years from listing. Younger patients less than 60 years of age had both significantly decreased waitlist and liver-related waitlist mortality when they had a pLD although the same benefit was not seen in older patients. There were no significant waitlist mortality or liver-related mortality benefits of pLD when stratified by body mass index (Appendix 1, http://links.lww.com/HC9/A428). The incidence of liver transplant did not differ significantly when patients had a pLD and were stratified by sex, age, or body mass index (Appendix 1, http://links.lww.com/HC9/A428).

### Exception points

**FIGURE 4:**
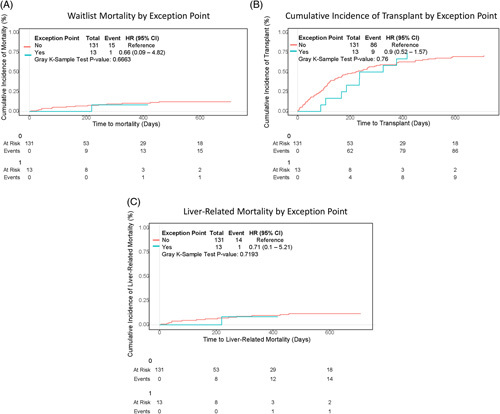
Outcomes by exception point from time to listing to date of death on the waitlist, date of last seen on the waitlist, or date of transplant. (A) Waitlist mortality with transplant as a competing risk (*p* = 0.67). (B) Cumulative incidence of transplant with death as a competing risk (*p* = 0.76). (C) Liver-related mortality with transplant and non–liver-related death competing risks (*p* = 0.72). Plots truncated at 2 years ensuring at least 10% of patients are still in the cohort.

Although many patients reported pruritus (58%, n = 78) and cholangitis (58%, n = 76), exception points were only granted to 9% (n = 13/144) patients based on recurrent cholangitis (n = 9) and medication-intractable pruritus (n = 2) (Table 1). None of the outcomes including overall mortality, cumulative incidence of liver-related mortality, and cumulative incidence of transplant differed by exception point status (*p* = 0.66, 0.72, and 0.76, respectively) (Figure 4 A–C).

### Time spent on the waitlist

Patients with PSC received LDLT more frequently (60%, 64/106) than DDLT (40%, 42/106). The median MELD-Na at transplant for those receiving LDLT was 20.0 (range: 8–36) compared to 25.5 (range: 12–40) for those receiving DDLT. In patients with high MELD-Na (equal or greater than 20), those who received LDLT a significantly greater portion spent <1.5 years on the waitlist compared to those who received DDLT (95% vs. 79%, *p* = 0.039). In patients with lower MELD-Na (<20), there was no significant difference in the proportion who spent less than 1.5 years on the waitlist between those who received LDLT and DDLT (84% vs. 100%, *p* = 0.20) (Table 3).

**TABLE 3:** Time on the waitlist (% <1.5 y) for PSC patients stratified by MELD-Na score

MELD score at time of transplant	PSC patients with living donor transplant	PSC patients with deceased donor transplant	Patients not transplanted^a^	
	Time on waitlist listing until transplant (n (%) <1.5 y)	Time on waitlist listing until transplant (n (%) <1.5 y)	Time on waitlist (n (%) <1.5 y)	Mortality on waitlist
MELD ≥ 20	37 (95) N = 39	26 (79) N = 33	250 (87.11) N = 287	115 (92.74) N = 124
MELD < 20	21 (84) N = 25	9 (100) N = 9	287 (77.57) N = 370	214 (40.15) N = 553
Overall	58 (90.3) N = 64	35 (83.3) N = 42	537 (81.74) N = 657	329 (49.92) N = 657

a All patients in the University Health Network data set listed from November 2012 to December 2019, with cutoff date March 31, 2021 from the entire University Health Network data set. Not exclusively PSC patients.

Abbreviations: MELD, model for end-stage liver disease; PSC, primary sclerosing cholangitis.

### Graft outcomes

After transplantation, 4 patients had nonanastomotic stricture concerning for recurrence of PSC, 4 had periods of acute cellular rejection, and 1 patient developed hepatic artery thrombosis eventually requiring retransplantation.

## DISCUSSION

Unlike most other liver injuries, once patients have end-stage disease, LT in PSC is the only treatment that prolongs life, given the ultimate failure of medical treatments to change the disease course.^20^ In our single-center North American cohort study of 172 patients with PSC referred for liver transplant assessment, we evaluated the clinical course, in the context of a mixed donor transplant program that includes access to live donation. Overall, patients with PSC and access to a pLD had higher rates of LDLT with decreased waitlist mortality. In our cohort, patients with PSC appeared dependent on access to LDLT to achieve successful desired outcomes after being waitlisted. This challenges the current allocation model, and challenges all liver units to consider how to offer equal access to living donor transplantation, a demonstrably life-prolonging procedure.

Patients who receive LT for PSC have excellent outcomes in comparison to non-PSC indications, with five-year survival rates of 75 to 86.4%.^7,21,22^ However, use of the MELD-Na criteria for transplant allocation has limitations and controversies. The MELD-Na score tends to under-represent the severity and extent of the disease in PSC compared to other forms of liver disease. Since the primary liver dysfunction in PSC manifests as elevation in bilirubin with comparatively lower creatinine and INR, patients will tend to present with a lower MELD-Na score despite being overtly unwell.^23^

The MELD-Na score may also systematically disadvantage patients with PSC because other important elements that affect patients with PSC, such as poor quality of life and increased risk of disease-specific adverse outcomes (recurrent or intractable cholangitis and biliary malignancies) are not captured.^24^ Consequently, wait times on transplant lists for patients with PSC have been typically longer than those for other indications^23^ as was also shown in this study, with an average time to transplant was 8.2 months. In comparison, the average time to transplant was approximately 5 months in our previous study for a broad range of transplant indications.^25^

This study shows that, as a whole and among subgroups, patients with PSC benefit from having a pLD in terms of greater overall survival, lower rate of liver-related mortality, and higher rate of transplant. Although a causative factor cannot be determined definitively, these findings suggest that having a pLD is associated with better outcomes. Patients with pLD have also been shown to have greater access to social capital (less likely to be divorced, single or having immigrant status) and financial support, and were more likely to be younger.^17^ Social determinants associated with DDLT included closer distance, populous areas, and lower household income.^16^ Access to LDLT may be especially important for women with PSC since women’s typically smaller size has disadvantaged their access to DDLT.^25^ Therefore, transplant programs incorporating LDLT, as well as transplant policy organizations, must be attuned to these potential equity challenges. The addition of sex in the new MELD 3.0 score may provide greater access to transplantation for women with PSC once implemented.^26^

A recent study by Jackson et al^27^ showed a survival benefit of 13–17 years in patients receiving LDLT compared to those who were not transplanted. In this study, 61% of patients transplanted for PSC received LDLT while 39% received DDLT. In comparison, in prior analyses at our center, only 24%–27% of transplants were LDLT across a wide range of indications.^16,25^ This rate of LDLT is also much higher than other indications for LT in the literature.^20^ In the post-MELD era, Goldberg et al^23^ found that US patients with PSC had 4 times greater odds of receiving LDLT than non-PSC patients. Other studies have also shown rates of LDLT that are several folds higher for PSC than for non-PSC indications (14% vs. 4%).^23^ This appears to be strong indirect evidence that patients and clinicians recognize that the current MELD-Na allocation system does not accurately reflect patients’ degree of illness or clinical need.

In this study, only a small minority (n = 13) of patients received exception points. This underscores the difficulty in meeting exception point criteria for PSC patients. Those who received exception points had an increased time to transplant. Patients with exception points were listed and transplanted at lower MELD-Na scores. Although the small sample size limits the analysis, this may suggest that patients with PSC who received exception points required these exception points to have clinical outcomes on par with those who did not require exception points. Unfortunately, our current data set does not support such an analysis and future studies need to be done. Understanding the effects of and the need for exception points for LT in PSC is important due to increasing controversy over their utility, given the overall low rate of waitlist mortality in these exception point patients, which was also demonstrated in this study.

There were several limitations to this study. First, only patients who were referred for transplant assessment were included, which means that the study group is selected for those with more severe diseases. There are many patients followed as outpatients who will have a long and relatively asymptomatic course, until the development of a severe complication (cholangitis and CCA), and thus may not have survived to be referred for transplant assessment. In addition, there was no comparator group of patients who did not have a diagnosis of PSC available. However, a recently published study from our center with approximately the same period of data collection was used to provide some context.^25^ Second, as this was a single-center study, and the decision for transplantation for PSC differs across the world, our experiences may not reflect those of other transplant centers. Finally, this study has the limitations inherent to a retrospective study, including some missing data.

## CONCLUSIONS

In this analysis, we demonstrate a higher rate and dependence on LDLT for people living with PSC than would be expected for other liver transplant indications and significantly increased survival and rates of transplant with access to pLD. We underscore the importance of access to LDLT for PSC in the current MELD-Na era, and highlight indirectly that patients with PSC are not adequately served with a pure MELD-Na approach. Future efforts are needed to redesign organ allocation approaches to better address the clinical needs for both common, as well as rare disease indications such as PSC. Equity of access to living donor transplantation needs to be enhanced to ensure equity of opportunity for a lifesaving procedure.

## Supplementary Material
