# Supplementary material for: Living donor liver transplantation can address disparities in transplant access for patients with primary sclerosing cholangitis
Source: Hepatol Commun. 2023 Aug 3;7(8):e0219. doi: 10.1097/HC9.0000000000000219 (PMC10552969; doi:10.1097/HC9.0000000000000219)
Supplement: Supplementary file 1 [file hc9-7-e0219-s001.pptx]

## Slide 1
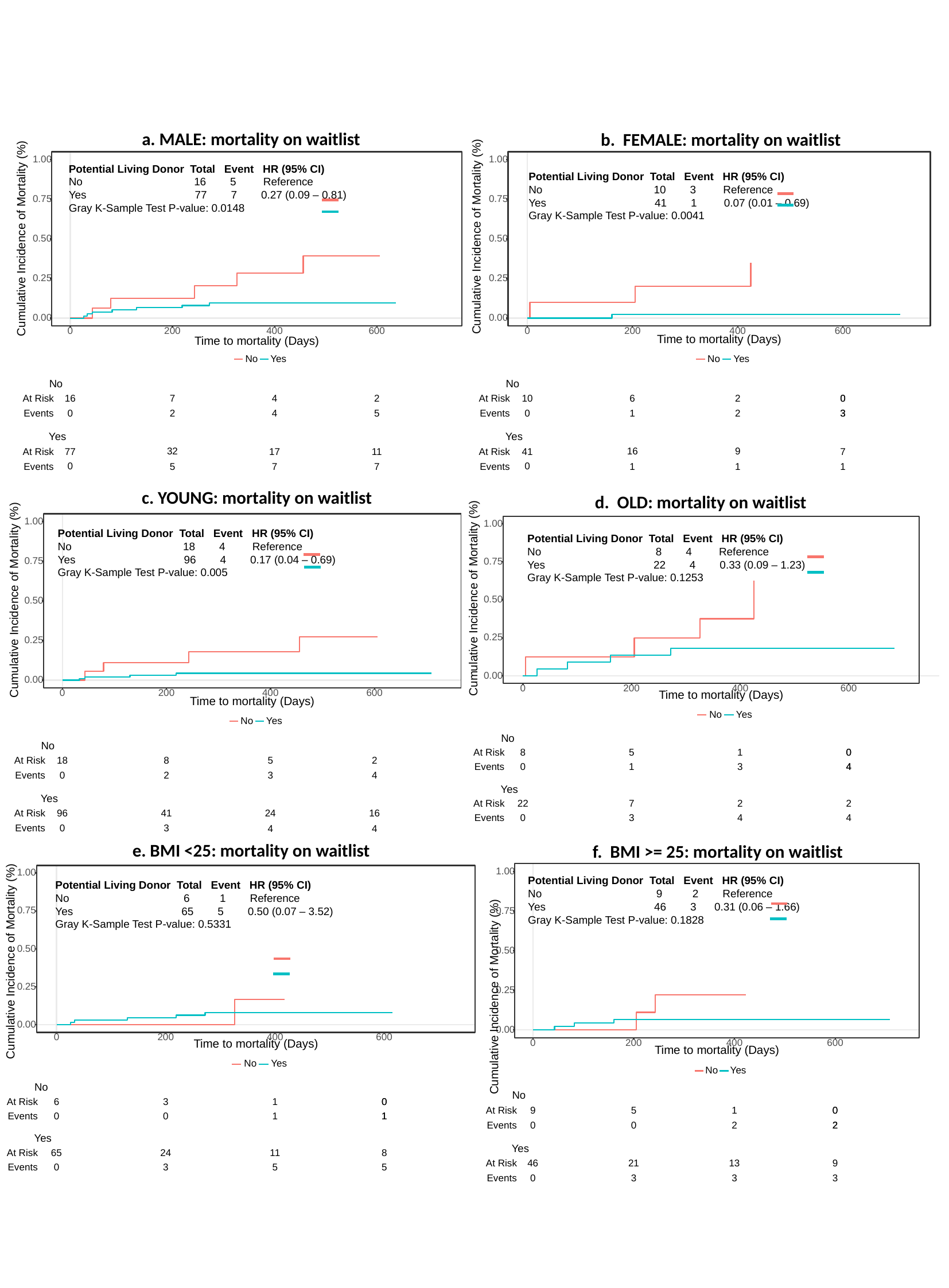

a. MALE: mortality on waitlist
b. FEMALE: mortality on waitlist
1.00
0.75
0.50
0.25
0.00
0
200
400
600
Time to mortality (Days)
No
Yes
No
16
At Risk
2
4
7
0
Events
2
5
4
Yes
32
At Risk
17
11
77
0
Events
5
7
7
1.00
0.75
0.50
0.25
0.00
0
200
400
600
Time to mortality (Days)
No
Yes
No
10
6
0
0
At Risk
2
3
3
0
Events
1
2
Yes
16
9
At Risk
41
7
0
Events
1
1
1
Potential Living Donor Total Event HR (95% CI)
No 16 5 Reference
Yes 77 7 0.27 (0.09 – 0.81)
Gray K-Sample Test P-value: 0.0148
Potential Living Donor Total Event HR (95% CI)
No 10 3 Reference
Yes 41 1 0.07 (0.01 – 0.69)
Gray K-Sample Test P-value: 0.0041
Cumulative Incidence of Mortality (%)
Cumulative Incidence of Mortality (%)
c. YOUNG: mortality on waitlist
d. OLD: mortality on waitlist
1.00
0.75
0.50
0.25
0.00
0
200
400
600
Time to mortality (Days)
No
Yes
No
18
8
At Risk
2
5
3
0
Events
2
4
Yes
96
16
At Risk
41
24
3
0
Events
4
4
1.00
0.75
0.50
0.25
0.00
0
200
400
600
Time to mortality (Days)
No
Yes
No
8
0
0
At Risk
1
5
3
0
Events
1
4
4
Yes
At Risk
22
2
2
7
3
0
Events
4
4
Potential Living Donor Total Event HR (95% CI)
No 18 4 Reference
Yes 96 4 0.17 (0.04 – 0.69)
Gray K-Sample Test P-value: 0.005
Potential Living Donor Total Event HR (95% CI)
No 8 4 Reference
Yes 22 4 0.33 (0.09 – 1.23)
Gray K-Sample Test P-value: 0.1253
Cumulative Incidence of Mortality (%)
Cumulative Incidence of Mortality (%)
e. BMI <25: mortality on waitlist
f. BMI >= 25: mortality on waitlist
1.00
0.75
0.50
0.25
0.00
0
200
400
600
Time to mortality (Days)
No
Yes
No
9
0
0
At Risk
1
5
0
0
Events
2
2
2
Yes
13
46
9
At Risk
21
3
3
3
0
Events
1.00
0.75
0.50
0.25
0.00
0
200
400
600
Time to mortality (Days)
No
Yes
No
3
6
0
0
At Risk
1
0
0
Events
1
1
1
Yes
65
8
At Risk
24
11
3
0
Events
5
5
Potential Living Donor Total Event HR (95% CI)
No 9 2 Reference
Yes 46 3 0.31 (0.06 – 1.66)
Gray K-Sample Test P-value: 0.1828
Potential Living Donor Total Event HR (95% CI)
No 6 1 Reference
Yes 65 5 0.50 (0.07 – 3.52)
Gray K-Sample Test P-value: 0.5331
Cumulative Incidence of Mortality (%)
Cumulative Incidence of Mortality (%)

## Slide 2
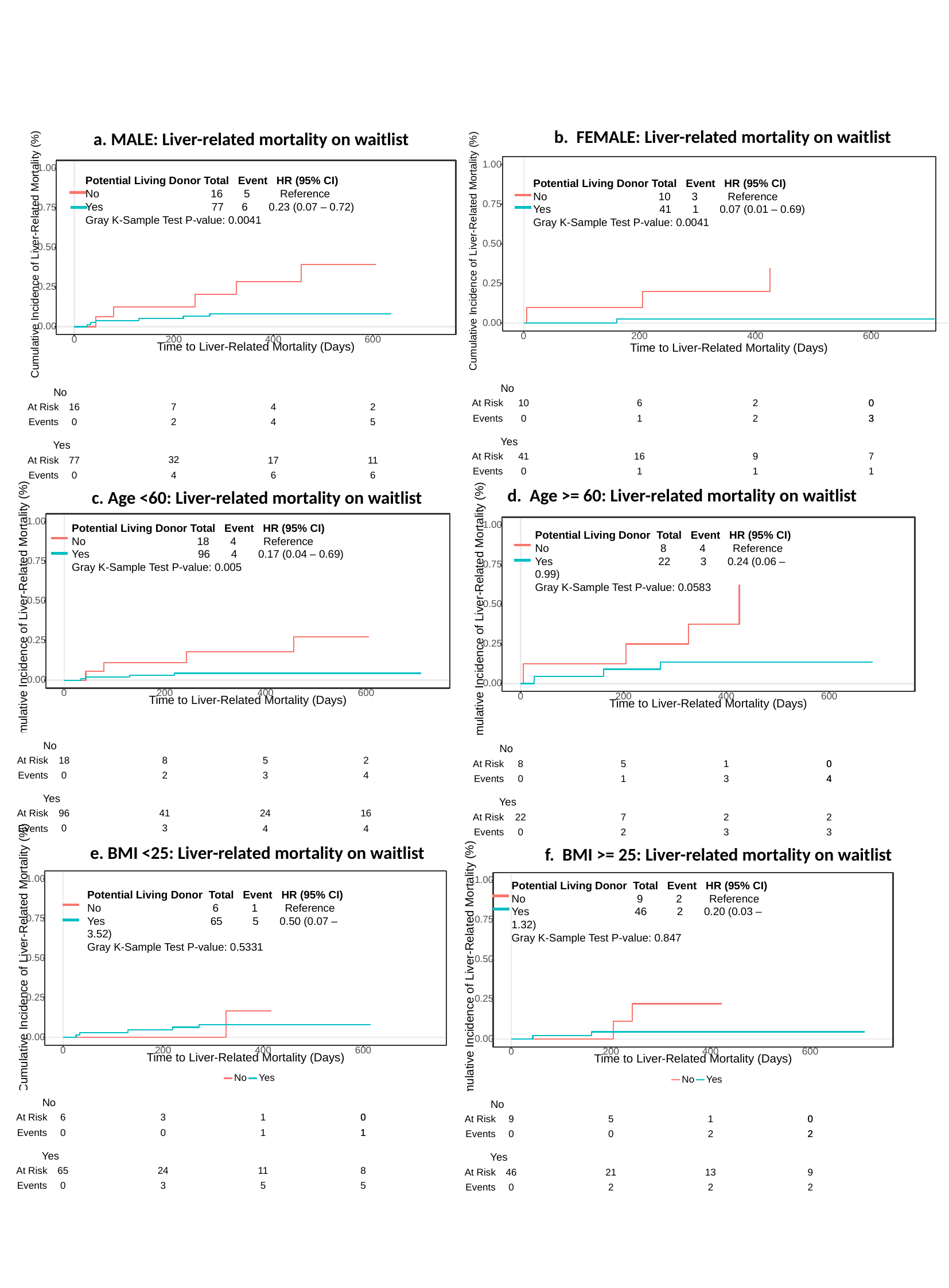

b. FEMALE: Liver-related mortality on waitlist
a. MALE: Liver-related mortality on waitlist
1.00
0.75
0.50
Cumulative Incidence of Liver-Related Mortality (%)
0.25
0.00
0
200
400
600
Time to Liver-Related Mortality (Days)
No
10
6
0
0
At Risk
2
3
3
0
Events
1
2
Yes
16
9
At Risk
41
7
0
Events
1
1
1
1.00
0.75
0.50
Cumulative Incidence of Liver-Related Mortality (%)
0.25
0.00
0
200
400
600
Time to Liver-Related Mortality (Days)
No
16
At Risk
2
4
7
0
Events
2
5
4
Yes
32
At Risk
17
11
77
0
6
6
Events
4
Potential Living Donor Total Event HR (95% CI)
No 16 5 Reference
Yes 77 6 0.23 (0.07 – 0.72)
Gray K-Sample Test P-value: 0.0041
Potential Living Donor Total Event HR (95% CI)
No 10 3 Reference
Yes 41 1 0.07 (0.01 – 0.69)
Gray K-Sample Test P-value: 0.0041
c. Age <60: Liver-related mortality on waitlist
d. Age >= 60: Liver-related mortality on waitlist
1.00
0.75
0.50
Cumulative Incidence of Liver-Related Mortality (%)
0.25
0.00
0
200
400
600
Time to Liver-Related Mortality (Days)
No
18
8
At Risk
2
5
3
0
Events
2
4
Yes
96
16
At Risk
41
24
3
0
Events
4
4
1.00
0.75
0.50
Cumulative Incidence of Liver-Related Mortality (%)
0.25
0.00
0
200
400
600
Time to Liver-Related Mortality (Days)
No
8
0
0
At Risk
1
5
3
0
Events
1
4
4
Yes
At Risk
22
2
2
7
3
3
0
Events
2
Potential Living Donor Total Event HR (95% CI)
No 18 4 Reference
Yes 96 4 0.17 (0.04 – 0.69)
Gray K-Sample Test P-value: 0.005
Potential Living Donor Total Event HR (95% CI)
No 8 4 Reference
Yes 22 3 0.24 (0.06 – 0.99)
Gray K-Sample Test P-value: 0.0583
e. BMI <25: Liver-related mortality on waitlist
f. BMI >= 25: Liver-related mortality on waitlist
1.00
0.75
Cumulative Incidence of Liver-Related Mortality (%)
0.50
0.25
0.00
0
200
400
600
Time to Liver-Related Mortality (Days)
No
Yes
No
3
6
0
0
At Risk
1
0
0
Events
1
1
1
Yes
65
8
At Risk
24
11
3
0
Events
5
5
1.00
0.75
0.50
Cumulative Incidence of Liver-Related Mortality (%)
0.25
0.00
0
200
400
600
Time to Liver-Related Mortality (Days)
No
Yes
No
9
0
0
At Risk
1
5
0
0
Events
2
2
2
Yes
13
46
9
At Risk
21
0
Events
2
2
2
Potential Living Donor Total Event HR (95% CI)
No 9 2 Reference
Yes 46 2 0.20 (0.03 – 1.32)
Gray K-Sample Test P-value: 0.847
Potential Living Donor Total Event HR (95% CI)
No 6 1 Reference
Yes 65 5 0.50 (0.07 – 3.52)
Gray K-Sample Test P-value: 0.5331

## Slide 3
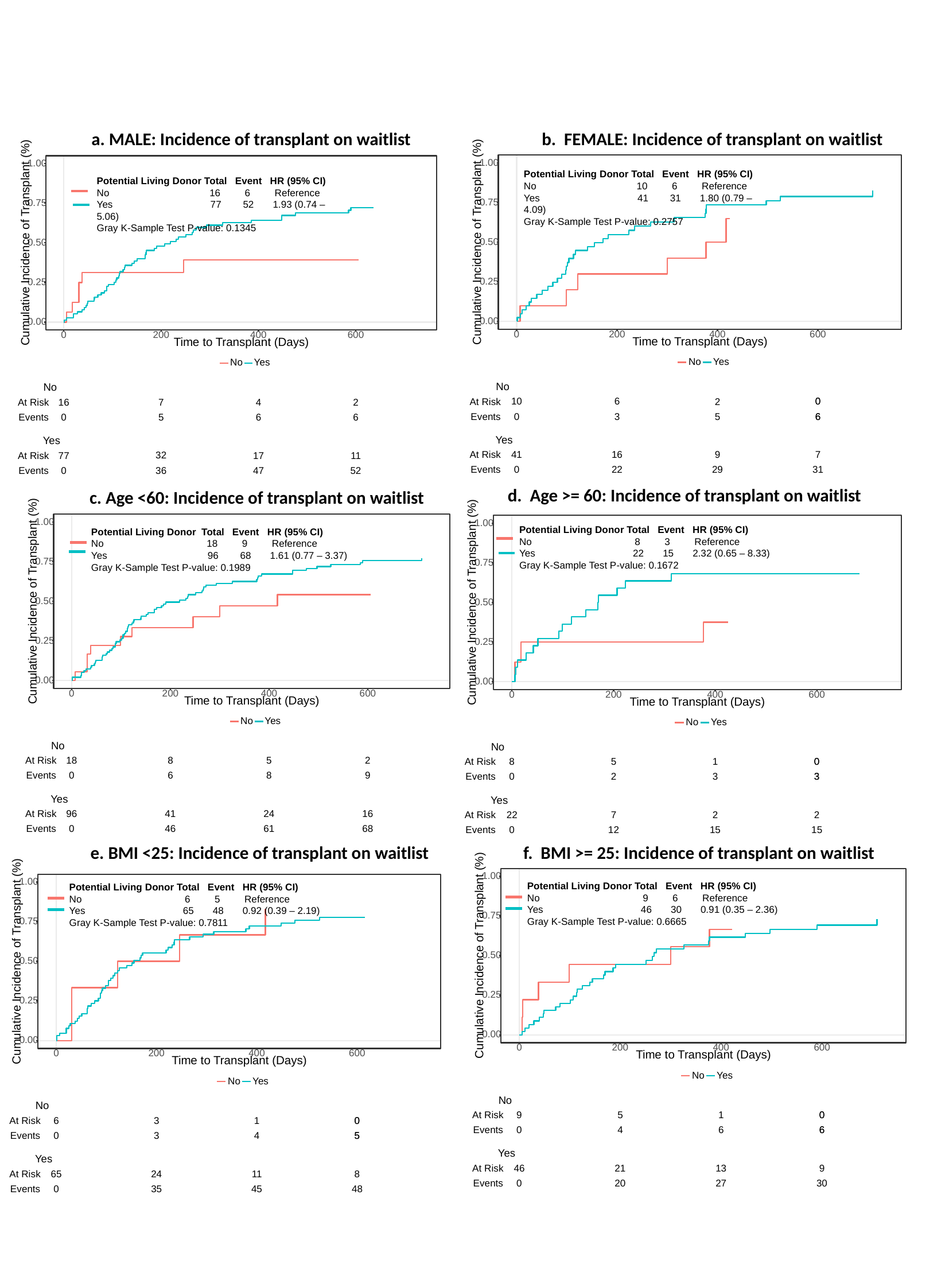

a. MALE: Incidence of transplant on waitlist
b. FEMALE: Incidence of transplant on waitlist
1.00
0.75
Cumulative Incidence of Transplant (%)
0.50
0.25
0.00
0
200
400
600
Time to Transplant (Days)
No
Yes
No
10
6
0
0
At Risk
2
3
0
6
6
Events
5
Yes
16
9
At Risk
41
7
31
0
29
Events
22
1.00
0.75
Cumulative Incidence of Transplant (%)
0.50
0.25
0.00
0
200
400
600
Time to Transplant (Days)
No
Yes
No
16
At Risk
2
4
7
0
6
6
Events
5
Yes
32
At Risk
17
11
77
36
0
52
Events
47
Potential Living Donor Total Event HR (95% CI)
No 10 6 Reference
Yes 41 31 1.80 (0.79 – 4.09)
Gray K-Sample Test P-value: 0.2757
Potential Living Donor Total Event HR (95% CI)
No 16 6 Reference
Yes 77 52 1.93 (0.74 – 5.06)
Gray K-Sample Test P-value: 0.1345
c. Age <60: Incidence of transplant on waitlist
d. Age >= 60: Incidence of transplant on waitlist
1.00
0.75
Cumulative Incidence of Transplant (%)
0.50
0.25
0.00
0
200
400
600
Time to Transplant (Days)
No
Yes
No
18
8
At Risk
2
5
0
6
8
9
Events
Yes
96
16
At Risk
41
24
0
46
61
68
Events
1.00
0.75
Cumulative Incidence of Transplant (%)
0.50
0.25
0.00
0
200
400
600
Time to Transplant (Days)
No
Yes
No
8
0
0
At Risk
1
5
3
3
3
0
Events
2
Yes
At Risk
22
2
2
7
0
15
15
Events
12
Potential Living Donor Total Event HR (95% CI)
No 8 3 Reference
Yes 22 15 2.32 (0.65 – 8.33)
Gray K-Sample Test P-value: 0.1672
Potential Living Donor Total Event HR (95% CI)
No 18 9 Reference
Yes 96 68 1.61 (0.77 – 3.37)
Gray K-Sample Test P-value: 0.1989
e. BMI <25: Incidence of transplant on waitlist
f. BMI >= 25: Incidence of transplant on waitlist
1.00
0.75
Cumulative Incidence of Transplant (%)
0.50
0.25
0.00
0
200
400
600
Time to Transplant (Days)
No
Yes
No
9
0
0
At Risk
1
5
0
6
6
6
Events
4
Yes
13
46
9
At Risk
21
30
0
20
Events
27
1.00
0.75
Cumulative Incidence of Transplant (%)
0.50
0.25
0.00
0
200
400
600
Time to Transplant (Days)
No
Yes
No
3
6
0
0
At Risk
1
3
0
Events
5
5
4
Yes
65
8
At Risk
24
11
35
0
48
Events
45
Potential Living Donor Total Event HR (95% CI)
No 9 6 Reference
Yes 46 30 0.91 (0.35 – 2.36)
Gray K-Sample Test P-value: 0.6665
Potential Living Donor Total Event HR (95% CI)
No 6 5 Reference
Yes 65 48 0.92 (0.39 – 2.19)
Gray K-Sample Test P-value: 0.7811
